# Image-Based Methods to Investigate Synchronization between Time Series Relevant for Plasma Fusion Diagnostics

**DOI:** 10.3390/e22070775

**Published:** 2020-07-16

**Authors:** Teddy Craciunescu, Andrea Murari, Ernesto Lerche, Michela Gelfusa, JET Contributors

**Affiliations:** 1 EUROfusion Consortium, JET, Culham Science Centre, Abingdon OX14 3DB, UK; 2 National Institute for Laser, Plasma and Radiation Physics, 077126 Măgurele, Romania; andrea.murari@euro-fusion.org (A.M.); Ernesto.Lerche@rma.ac.be (E.L.); gelfusa@ing.uniroma2.it (M.G.); 3 Consorzio RFX (CNR, ENEA, INFN, Universita di Padova, Acciaierie Venete SpA), I-35127 Padova, Italy; 4 EUROfusion Programme Management Unit, JET, Culham Science Centre, Abingdon OX14 3DB, UK; 5 LPP-ERM/KMS, Association EUROFUSION-Belgian State, TEC Partner, 1000 Brussels, Belgium; 6 Associazione EUROfusion—University of Rome “Tor Vergata”, Via Orazio Raimondo, 18, 00173 Roma, Italy

**Keywords:** Gramian angular field, Markov transition field, chaos game representation, complex networks, entropy, sawteeth, pacing experiments, tokamaks

## Abstract

Advanced time series analysis and causality detection techniques have been successfully applied to the assessment of synchronization experiments in tokamaks, such as Edge Localized Modes (ELMs) and sawtooth pacing. Lag synchronization is a typical strategy for fusion plasma instability control by pace-making techniques. The major difficulty, in evaluating the efficiency of the pacing methods, is the coexistence of the causal effects with the periodic or quasi-periodic nature of the plasma instabilities. In the present work, a set of methods based on the image representation of time series, are investigated as tools for evaluating the efficiency of the pace-making techniques. The main options rely on the Gramian Angular Field (GAF), the Markov Transition Field (MTF), previously used for time series classification, and the Chaos Game Representation (CGR), employed for the visualization of large collections of long time series. The paper proposes an original variation of the Markov Transition Matrix, defined for a couple of time series. Additionally, a recently proposed method, based on the mapping of time series as cross-visibility networks and their representation as images, is included in this study. The performances of the method are evaluated on synthetic data and applied to JET measurements.

## 1. Introduction

The diagnostic systems monitoring the experiments in Magnetic Confinement Nuclear Fusion (MCNF) produce a large amount of data, several tens of Gigabytes for a typical Joint European Torus (JET) experiment, mainly in the form of time series. Discovering temporal causal directions or assessing the causal relations between dynamical processes, represented as time series, is an important task in various fusion plasma studies. The investigation of instabilities and the assessment of the pacing techniques efficiency, the study of L–H transitions, for which a theoretical dynamical model is not yet available, or the study of plasma dynamics in relation to the impurity control are significant examples.

The control of major instabilities such as Edge Localized Modes (ELMs) and sawteeth [1], with a significant potentially harmful impact on the devices, represents a key issue for the development of the next large fusion devices such as ITER (International Thermonuclear Experimental Reactor). Various forms of pacing, based on external perturbations, have been proposed. In the case of ELMs, the periodic injection of small frozen pellets of fusion fuel into the plasma edge at high frequency is a very promising solution [2]. The modulation of the Ion Cyclotron Radiofrequency Heating (ICRH) power has proven to be an efficient solution for sawteeth pacing, reducing the fast ion component, which has a stabilizing effect [3]. Pellets and ICRH notches represent time localized perturbations of the natural evolution of the plasma, with a clear causal direction. On the other hand, the instabilities are quasiperiodic in nature and, therefore, if enough time is allowed to elapse, they are bound to reoccur, making difficult the interpretation of the experimental data. To evaluate the effectiveness of the triggering capabilities of the pacing techniques, it is, therefore, necessary to determine the time interval when the pacing has a causal influence on the instability dynamics.

The study of time series, generated by delayed coupled dynamical systems, is a very difficult problem, far from being completely solved in its full complexity [4,5]. Frequently the performances of the methods, evaluating the time series coupling, may vary depending on the type of the dynamic systems studied. In the case of fusion plasma instability pacing, some physics properties render the problem more tractable: the coupling is unidirectional and it may be assumed that its strength is relatively strong, lying in the domain where these kinds of techniques are more sensitive. Several methods such as Granger Causality, Transfer Entropy, Recurrence Plots, Convergent Cross Mapping have been successfully applied for the evaluation of the time-lagged causal relations between time series, related to the instabilities and pacing factors [6,7,8]. 

**Figure 1:**
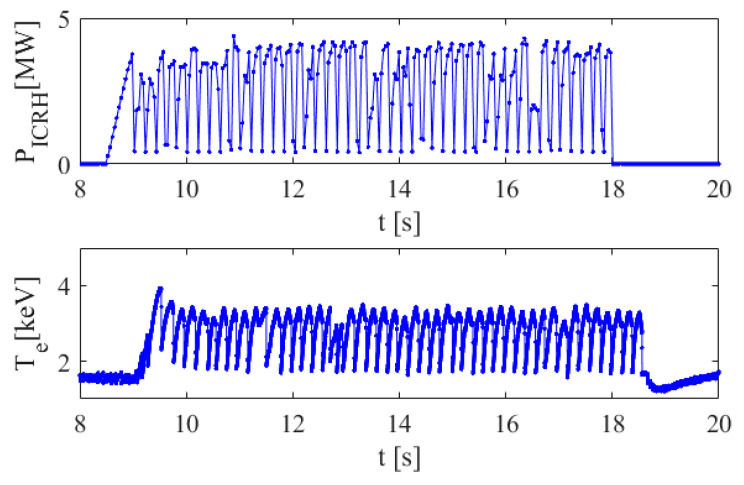
An example of the time series obtained in sawteeth pacing with Ion Cyclotron Radiofrequency Heating (ICRH) modulation in the JET-ILW L mode discharge #89826. The ICRH power time series is presented in the top plot while the central electron temperature, influenced by the sawteeth, is shown in the bottom plot. The frequency of the modulation is 5 Hz (150 ms on and 50 ms off). The maximum power is 4 MW in a minority heating scheme with 4% of H in D. The sampling frequency in the sawteeth time series is 10^−6^ s.

In this paper, the efficiency of a group of methods, based on the time series representation as images, is investigated. The methods are evaluated on synthetic data and then on the JET experimental data. We are using the same experimental data as in [7] in order to allow a comparison of the methods. The measurements have been performed during sawteeth pacing experiments with ICRH modulation in JET–ITER-like Wall (JET-ILW) L and H mode discharge. A typical example is presented in Figure 1. The reader is referred to [3,7] for a detailed description of the pacing technique.

The paper is organized as follows: the next section gives a brief overview of the methods for time series encoding as images and a more detailed description of the techniques used in this paper. Section 3 is devoted to the evaluation of the methods’ performances on synthetic data. An application to experimental measurements in thermonuclear fusion is also described in this section. Several conclusions are drawn in the last section of the paper.

## 2. Image Representation of Time Series

Encoding time series as images have emerged in the field of data mining as an alternative tool for measuring similarities. The great success of deep learning in computer vision has been also a factor in promoting the representation of time series as different types of images for visualization, allowing a quick overview of large datasets and the easy discovery of interesting patterns, anomalies, gaps, clusters, etc. It has to be noted that, even when the analysis process is not fully computerized, the human perceptual skills are able, in the case of adequate representations, to cope with such visual tasks in a few hundred milliseconds [9]. The image representation has also the advantage of greater scalability to larger datasets. A simple intuitive representation, capable of describing complex patterns of repetition in time series, has been introduced in [10]. The data is converted to string representation, by mean of a discretization technique, and arcs are drawn between identical string sequences. A different approach to pattern discovery has been introduced in [11]. The data is discretized in the same manner and the symbolic representation is encoded in a modified suffix tree, where each pattern corresponds to a branch of the tree. The frequency of the patterns is mapped into a property of the graph (usually color or thickness). The visualization of periodic time series data based on spirals has been introduced in [12]. 

More popular approaches, used also in the present paper, are the Gramian Angular Fields (GAF) and Markov Transition Fields (MTF) methods [13], adequate choices when using Convolutional Neural Networks (CNN) for time series classification, and the Chaos Game Representation (CGR), which allows a synthetic and compact representation of large time series [14]. A recently developed method, based on the transformation of time series in a complex network and the image entropy evaluation of a modified adjacency matrix [15], is also included in this study. These techniques are presented in the following subsections.

Other promising approaches have been proposed recently. A time series imaging encoding scheme called Motif Difference Field (MDF), based on the motifs of different lengths, has been described in [16]. The approach is particularly useful for time series clustering allowing higher-order patterns or structures discovery in time series data. The embedding of Recurrence Plots (RP) [17] in the Bag of Features (BoF) [18] model, which summarizes time series according to the frequencies of ”feature words” of a data-learned dictionary, has been documented in [19]. In this way, advanced image processing techniques (such as, e.g., Scale-invariant feature transform (SIFT) [20]), Histogram of Oriented Gradients (HOG) [21] and Local Binary Patterns (LBP) [22]) are integrated with the time series analysis. An alternative data representation scheme is introduced in [23]. Time series are transformed into a two-dimensional real-coordinate space of amplitude and first- or higher-order derivatives. 

### 2.1. Gramian Angular Field (GAM)

The Gramian Angular Field (GAF) method represents the time series $X=\left\{x_{1,\;}x_{2,\;}\ldots ,x_{n,\;}\right\}$, after rescaling it in the interval [−1, 1], on a polar coordinate:

(1) $$\{\begin{array}{c} \varphi =\arccos\left(x_{i}\right),-1<x_{i}<1\\ r=\frac{t_{i}}{N}\; \end{array}$$

where $t_{i}$ is the time stamp and N is a constant introduced to regularize the span of the polar coordinate system. Depending on the evolution of the time series, the shape formed by the points defined in (1) twists at specific angular points, creating a unique encoding map, which preserves absolute temporal relations [13]. GAF images are represented as a Gramian matrix where each element is the trigonometric sum of different time intervals:

(2) $$G=\left[\begin{array}{ccc} cos\left(\varphi_{1}+\varphi_{1}\right) & \cdots & cos\left(\varphi_{1}+\varphi_{n}\right)\\ \vdots & \ddots & \vdots \\ cos\left(\varphi_{n}+\varphi_{1}\right) & \cdots & cos\left(\varphi_{n}+\varphi_{n}\right) \end{array}\right]$$

Each element $G_{k}$ represents the relative correlation by superposition of directions with respect to the time interval *k*. 

It may be assumed that, when synchronization progresses, the GAF images of the driver and the target will have an increased number of common features, leading to an increased degree of similarity. The GAF images similarity may be evaluated by mean of the Structural Similarity Index (SSIM) [24]. SSIM is a very popular metric that was originally introduced to quantify image quality degradation during various processes such as, e.g., compression or data transmission. It is considered to be correlated with the human visual system quality perception. SSIM models the image degradation based on a combination of luminance and contrast distortion plus the loss of correlation. It is defined by the relation:

(3) SSIM(f,g)=l(f,g)·c(f,g)·s(f,g)

where:

- $l\left(f,g\right)=\frac{2\mu_{f}\mu_{g}+C_{1}}{\mu_{f}{}^{2}+\mu_{g}{}^{2}+C_{1}}$ is the luminance comparison term, $\mu_{f}$, $\mu_{g}$ are the luminances of images f and g. This term is equal to one for identical luminaces.
- c$\left(f,g\right)=\frac{2\sigma_{f}\sigma_{g}+C_{2}}{\sigma_{f}{}^{2}+\sigma_{g}{}^{2}+C_{2}}$ is the contrast comparison term, the contrast is measured by the standard deviation $\sigma_{f}$, $\sigma_{g}$ of images f and g.
- s$\left(f,g\right)=\frac{\sigma_{fg}+C_{3}}{\sigma_{f}\sigma_{f}+C_{3}}$, is the structure comparison term, measured by the correlation coefficient between the two images f and g, $\sigma_{fg}$ is the covariance between f and g.

The positive constants $C_{i},\;i=1,2,3$ are introduced in order to avoid a null denominator. Typical choices are: $C_{1}={\left(0.01\cdot L\right)}^{2}$, $C_{2}={\left(0.03\cdot L\right)}^{2}$ and $C_{3}=\frac{C_{2}}{2}$, where L is the dynamic range of the image element’s values. SSIM values lie in the interval [0, 1]

### 2.2. Markov Transition Field (MTF)

The idea of encoding the dynamical transition statistics has been exploited first by Campanharo et al. [25], who proposed the construction of a complex network, based on the transition probability in a Markov model. First, a number of Q quantile bins are identified and then each value of the time series $x_{i}\in X\;$ is assigned to a specific bin q∈[1,…,Q]. Each bin represents a node in the network and two nodes are connected with a weight proportional to the probability that a point in quantile $q_{i}$ is followed by a point in quantile $q_{j}$ in the time series. The corresponding adjacency matrix is given by the relation:

(4) $$W=\left[\begin{array}{ccc} W_{11|P(x_{t}\in q_{1}|P\left(x_{t-1}\in q_{1}\right)} & \ldots & W_{1Q|P(x_{t}\in q_{1}|P\left(x_{t-1}\in q_{Q}\right)}\\ \begin{array}{c} .\\ .\\ . \end{array} & \begin{array}{c} .\\ .\\ . \end{array} & \begin{array}{c} .\\ .\\ . \end{array}\\ W_{Q1|P(x_{t}\in q_{Q}|P\left(x_{t-1}\in q_{1}\right)} & \ldots & W_{QQ|P(x_{t}\in q_{Q}|P\left(x_{t-1}\in q_{Q}\right)} \end{array}\right]$$

where $w_{ij}$ gives the frequency with which a point in quantile $q_{j}$ follows a point in quantile $q_{j}$. As shown in [25] an approximate inverse of the mapping W can be retrieved.

The Markov matrix W, incorporates the Markov dynamics but, on the other hand, discards the conditional relationship between the distribution of X and the temporal dependency on the time steps $t_{i}$. In order to avoid this information loss, Wang et al. [13] proposed an extension of the Markov matrix by aligning each probability along the temporal order:

(5) $$MTF=\left[\begin{array}{ccc} w_{ij|x_{1}\in q_{i},x_{1}\in q_{j}} & \ldots & w_{ij|x_{1}\in q_{i},x_{n}\in q_{j}}\\ \begin{array}{c} .\\ .\\ . \end{array} & \begin{array}{c} .\\ .\\ . \end{array} & \begin{array}{c} .\\ .\\ . \end{array}\\ w_{ij|x_{n}\in q_{i},x_{1}\in q_{j}} & \ldots & w_{ij|x_{n}\in q_{i},x_{n}\in q_{j}} \end{array}\right]$$

where $MTF_{ij}$ represents the transition probability from the quantile at $t_{i}$ to the quantile at $t_{j}$, which now are not necessarily consecutive. Therefore, MTF encodes the multispan transition probabilities of the time series. For example, $MTF_{i,j,\left|i-k\right|=k}$ represents the transition probability between the points separated by the temporal distance k. MTF is a unique representation for a fixed value of Q. However, the mapping is surjective and, therefore, it is not possible to recover the time series from MTF. 

A MTF mapping can be created for each time series. In principle, it may be assumed that the similarity of the images will increase with the increase of the coupling between the time series. This assumption is tested by numerical examples in the next section of this paper. The SSIM factor can be again used as a similarity measure.

For a pair of coupled time series, we are proposing, for the first time in this paper, to calculate the Markov matrix (4) for a new time series, created by intercalating the values of the two time series:

(6) $$\{\begin{array}{c} z_{2\cdot i-1}=x_{i}\\ z_{2\cdot i}=y_{i} \end{array},\;i=1,\ldots ,N$$

**Figure 2:**
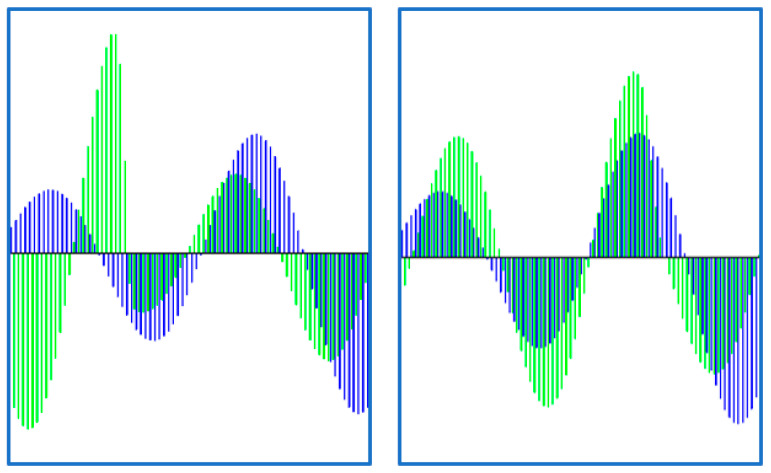
Illustration of the construction of a new time series by the intercalated merge of a pair of time series. The graphs have been obtained using a Rössler coupled system and two different values of the coupling parameter. The driver is the blue curve and the target the green one. In the case of the left plot the coupling is zero, while in the right plot the coupling coefficient is set to C = 0.5.

The intercalating technique described by (6) is illustrated in Figure 2. The corresponding matrix will be called the Cross Markov Matrix (CMM) and it will be denoted by $W_{cross}$. $W_{cross}$ has an expression similar to Equation (4). Its elements $w_{ij}^{cross}$ represent the frequency with which a point from the time series X in the quantile $q_{i}^{X}$ is followed by a point from the time series Y, in the quantile $q_{j}^{Y}$. For synchronized time series it may be expected that the transition between distant quantiles will diminish creating a specific pattern. The matrix $W_{cross}$ can be represented as an image and its evolution towards a more organized structure can be monitored by the image entropy:

(7) $$H=-\sum_{k}p_{k}\log_{2}p_{k}$$

where, in general, k is the gray level index and $p_{k}$ is the probability associated with gray level k.

An increased coupling between time series is expected to translate into a lower value of the entropy H. 

### 2.3. Chaos Game Representation (CGR)

The Chaos Game Representation (CGR) has been used for representing time series as a collection of thumbnails, allowing the fast and easy exploration and classification of large data sets [14]. CGR is an algorithm for producing pictures of fractal structures [26]. It is mathematically described by an iterated function system (IFS). An IFS is a set of affine maps $\left\{w_{i}\right\}$, where each map consists of a pair of linear equations:

(8) w(x,y)=(ax+by+e, cx+dy+f)

A certain probability is associated to map $w_{i}$. The chaos game starts by generating a random point in the unit square. A map $w_{k}$ is randomly chosen from the set $\left\{w_{i}\right\}$, according to its probability. Then, $w_{k}$ is applied to the current point to generate the next one. Different parameters of the IFS lead to different fractals. 

A particular simple version of the algorithm (CGRS) starts by fixing a number of vertices $N_{v}$ and by generating a starting random point $\left(x_{0},\;y_{0}\right)$. Then, at each step, one of the vertices $N_{k}$ is chosen randomly and the next point $\left(x_{k+1},\;y_{k+1}\right)$ is located halfway between the current point $\left(x_{k+1},\;y_{k+1}\right)$ and $N_{k}$. When running CGR using a truly random generator, all the points in the geometrical shape defined by the vertices will be visited. However, if CGRS is run using a nonrandom sequence of numbers, which can be associated intuitively with a certain structure, then the visual representation will display some underlying structure in the sequence of numbers. 

The data feeding the CGRS algorithm must be represented on an alphabet whose size should be equal to the number of vertices $N_{v}$. Therefore, the real-valued time series should be converted into discrete symbols. A popular technique is the SAX algorithm introduced by Lin et al. [27], widely used in data mining due to the dimensionality reduction carried over the symbolic representation. SAX also allows defining a lowest bound distance measure for the original series. The SAX algorithm uses a sliding window circulating on the time axis and a division of the ordinate axis in a certain number of intervals. For each position of the window, the values of the time series are replaced by the mean values corresponding to the current position of the sliding window. 

The output of the CGRS method consists of a graph $P\equiv \left\{x_{i},y_{i}\right\}$ located in the spatial domain $D\equiv \{0<x_{i}<x_{max};\;0<y_{i}<y_{max}\}$. The set of points P can be transformed into an image Im by superimposing a rectangular grid of size N×N on the domain D. The value of each image pixel is equal to the number of points $\left\{x_{i},y_{i}\right\}\in P$ falling inside the limits defining the image pixel (i,j). Then images corresponding to different time series can be compared by means of an appropriate image similarity measure. An alternative approach is to use a graph similarity measure for comparing graphs corresponding to different time series. 

The problem of deriving graph similarity measures is of key importance in structural pattern recognition. An efficient measure, the Symmetrized Normalized-Entropy-Square Variation (SNESV), has been proposed in [28]. For two graphs X, Y (or, in general, for two low-dimensional manifolds resulting from the commute time embedding of graphs $\Theta_{X},{\;\Theta }_{Y}$) the SNESV is defined by the relation:

(9) $$SNESV\left(\Theta_{X},\Theta_{Y}\right)=\frac{{\left(H\left(\Theta_{Y}\right)-H\left(\Theta_{X}\right)\right)}^{2}}{H\left(\Theta_{Y}\right)+H\left(\Theta_{X}\right)}=\frac{{\left(H\left(\Theta_{Y}\right)-H\left(\Theta_{X}\right)\right)}^{2}}{I\left(\Theta_{Y},{\;\Theta }_{X}\right)+H\left(\Theta_{Y},\Theta_{X}\right)}$$

where H(.), H(.,.) are the Shannon and the joint entropy, respectively, and I(.,.) is the mutual information. The normalization by the sum of entropies is important when comparing graphs with a significantly different number of nodes.

In practical applications, the estimation of information quantities (entropy, mutual information, etc.) should be performed from the available samples. The samples are supposed to be i.i.d. drawn from a distribution that is unknown. One possible approach is to assume a certain form of the underlying distribution and to derive its parameters from the experimental samples. The strong and often questionable assumption, about the form of the probability distribution function, can be avoided by nonparametric approaches. A popular choice, initially proposed by Kozachenko and Leonenko [29], is the method based on the k-nearest neighbors (kNN) techniques [30]. Considering a graph X and the probability density $p_{X}\left(X\right)$, the basic idea is to estimate the unknown $p_{X}\left(x_{i}\right)$ through the k-nearest neighbors (kNN) of $x_{i}$. Then the probability density and the subsequent quantities of interest are estimated based on distances (in an appropriate norm) of the samples to their k-nearest k-NN neighbors (see, e.g., [31] for a detailed example). The method proved to work well for a small fixed k (typically in the range of 4÷8) [32] providing significantly better results in comparison with other approaches like, e.g., kernel density estimator (KDE) [33] or entropy estimation using spacings [34].

An optimal choice for the estimation of SNESV is the kNN-based bypass estimator proposed by Leonenko [35], which provides the following expression of the entropy for SNESV [28]:

(10) $${\hat{H}}_{N,k}=\frac{1}{N}\overset{N}{\underset{i=1}{\sum }}log\left\{\left(N-1\right)e^{-\Psi \left(k\right)}V_{d}{\left(\rho_{k,N-1}^{\left(i\right)}\right)}^{d}\right\}$$

where:

- *N* is the number of samples $\left\{x_{1},\ldots ,x_{N}\right\}\equiv X\in R^{d}$.
- k the maximum number of nearest neighbors.
- Ψ(k) is the digamma function: $\Psi \left(k\right)=-\gamma +A_{k-1},\;\gamma =0.5772$ (Euler constant) $A_{0}=0,\;A_{j}=\overset{j}{\underset{i=1}{\sum }}\frac{1}{i},V_{d}=\pi^{\frac{d}{2}}/\Gamma \left(\frac{d}{2}+1\right)$ is the volume of the unit ball in $R^{d}$ and $\rho_{k,N-1}^{\left(i\right)}$ is the k-th nearest neighbor distance from $x_{i}$ to some other $x_{j}$.

Leonenko’s estimator has the advantage of fast computation. 

### 2.4. Complex Networks (CN)

The cross-visibility (CV) networks [36] extends the applicability of visibility graphs (VG) [37] to the study of coupled time series. VGs are based on the representation of time series by vertical bars, which create a landscape of peaks and valleys. Each bar corresponds to a node in the network and two nodes are connected if the corresponding bars are visible by each other, through the obstacles created by the other bars in the landscape. For the case of two time series, the CV method allows the creation of a link between nodes if the corresponding bars are reciprocally visible through the obstacles created by the shifted time series $\left\{y_{k}\right\}=\left\{y_{k}-y_{i}+x_{i}\right\}\;$[36]:

(11) $$y_{k}\leq y_{i}+\frac{x_{j}-x_{i}}{j-i}\left(k-i\right),\;\;\;\;i<\forall k<j$$

or

(12) $$y_{k}\geq y_{i}+\frac{x_{j}-x_{i}}{j-i}\left(k-i\right),\;\;\;\;i<\forall k<j$$

A weighted adjacency matrix WAM can be created for this network:

(13) $$WAM_{ij}=\{\begin{array}{c} \;d\left(y_{i},\;y_{j}\right),\;if\;Equations\;\left(18\right)\;or\;\left(19\right)\;are\;satisfied\\ 0,\;otherwise \end{array}$$

where $d\left(y_{i},\;y_{j}\right)$ is a similarity measure, between the time series segments located in between the connected nodes, for weighting the connections. It is calculated based on $L_{p}$ norms [38,39]:

(14) $$d_{L_{p}}{\left(x,y\right)}_{\left[i↔j\right]}={\left(\overset{j}{\underset{k=i}{\sum }}{\left(x_{k}-y_{k}\right)}^{p}\right)}^{\frac{1}{p}}$$

As shown in [15,40], WAM can be represented as an image and used for monitoring the complexity of the network, which is linked to the degree of coupling between time series. The image entropy H can be used to evaluate the complexity of the WAM image. For an independently evolving time series, the reciprocal visibility between two bars (corresponding to two components of the time series) is frequently limited by the obstacles created by the other time series. When coupled, the time series lean towards synchronization and the WAM image evolves toward a less random structure, characterized by lower entropy $E_{WAM}$.

## 3. Results and Discussion

### 3.1. Numerical Tests

**Figure 3:**
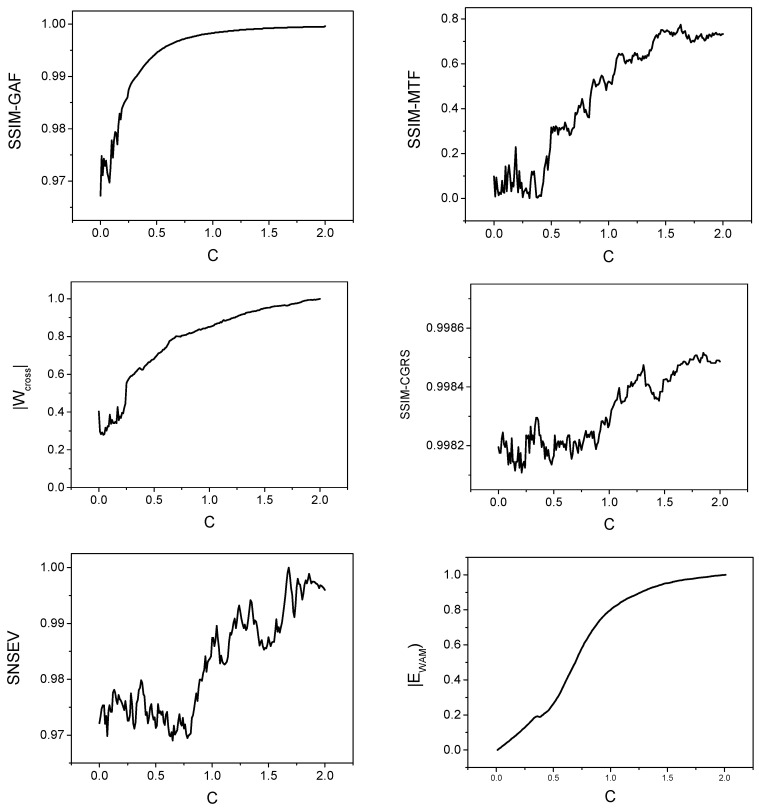
The evolution of the coupling measures with the coupling strength for the Rössler system: Structural Similarity Index (SSIM) calculated for Gramian Angular Field (GAF) images (top-left), SSIM calculated for Markov Transition Field (MTF) images (top-right), absolute value of the $W_{cross}$ image entropy (middle-left), SSIM calculated for the CGRS images (middle-right), SNSEV entropy for the CGRS graphs (bottom-left) and absolute value of the WAM image entropy (bottom-right). The absolute values of the entropies (SNSEV, $E_{cross}$ and $E_{WAM}$) are normalized to their maximum value.

The evaluation of the efficacy of the coupling measures, based on the image representation of time series, has been performed by using the unidimensional coupled Rössler system [41,42], which generates time series characterized by a succession of regular shaped peaks, similar to those recorded in plasma instability pacing experiments. The Rössler system, frequently used in the study of dynamic coupled systems (see, e.g., [43,44]), is described by the relations:

(15) $$\dot{x_{1}}=-0.95x_{2}-x_{3}\;\dot{x_{2}}=0.95x_{1}+0.15x_{2}\;\dot{x_{3}}=0.2+x_{3}\left(x_{1}-10\right)\;\dot{y_{1}}=-1.05y_{2}-y_{3}+C\left(x_{1}-y_{1}\right)\;\dot{y_{2}}=1.05y_{1}+0.15y_{2}\;\dot{y_{3}}=0.2+y_{3}\left(y_{1}-10\right)\;$$

where C describes the coupling strength. The time series $x_{2}$ and $y_{2}$ have been used as input to the various methods. We have used SSIM index for GAF (Equation (2)), MTF (Equation (6)) and CGRS images as a coupling measure for the corresponding time series. SNSEV entropy has been used as an alternative measure in the case of CGRS represented as graphs, while the image entropy has been used for the newly introduced CMM and also for monitoring the evolution of the structure of the CV networks. The evolution of these measures when increasing the coupling factor C in the interval [0, 2], in steps of 0.01, is presented in Figure 3. We used 10,000 data points to compute the image representations and to evaluate the dependence on the coupling strength of the above-mentioned measures.

**Figure 4:**
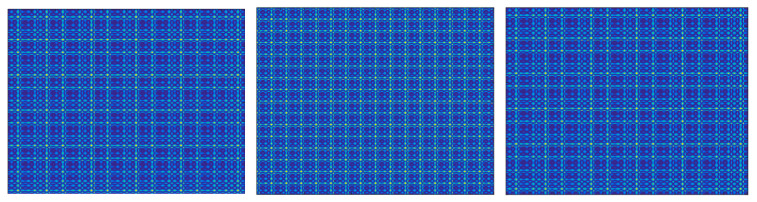
GAF image of the driver time series $x_{2}$ (left) and of the driven time series $y_{2}$ for C=0 (middle) and C=2 (left).

**Figure 5:**
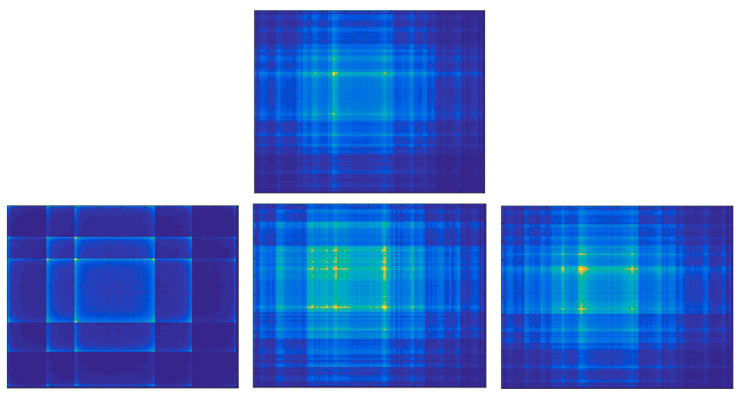
MTF image of the driver time series $x_{2}$ (top) and of the driven time series $y_{2}$ for C=0, 0.65, 1.35 (bottom, from left to right).

**Figure 6:**
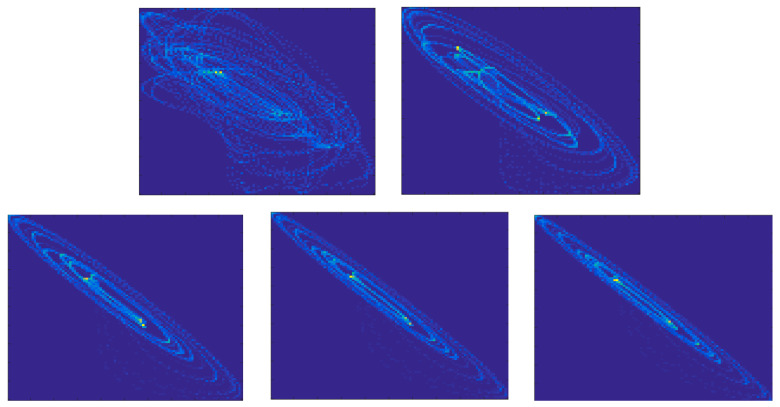
The evolution of the $W_{cross}$ images with the coupling parameter. Top row show the images for C=0 (left) and C=0.5 (right) and the bottom row shows the values for C=1.0 (left), C=1.5 (middle) and C=2 (right).

SSIM values calculated for the GAF representation lies on a quite limited range; however, the evolution with the coupling parameter is monotonic for C>0.15. The limited range of values can be explained by the structure of GAF images (Figure 4). Even for the case when the time series are not coupled (C=0) the images have a similar general structure, the differences lying at the level of details. For the same reason, the SSIM evolution reaches a saturation effect for C>1. In the case of MTF representation, the values cover a wider range but the evolution is characterized by various oscillations. The images corresponding to the driven time series (Figure 5) varies substantially with the variation of the coupling parameter. The MTF images have been obtained for a number of Q=100 quantiles. The evolution of the absolute values of the Cross Markov Matrix entropy is characterized by both monotonicity (for C>0.22) and by a wide range of variation and, therefore, it may be a good candidate for studying the lag coupling of fusion relevant time series. The evolution of the $W_{cross}$ images towards a more structured form, with lower entropy, is illustrated in Figure 6.

**Figure 7:**
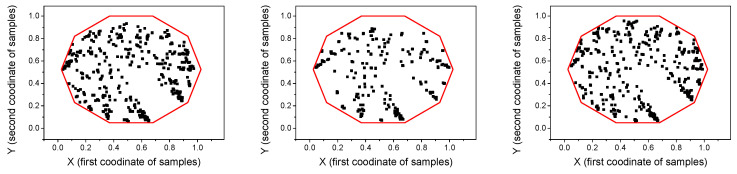
CGRS graph for the driver time series $x_{2}$ (left) and for the driven time series $y_{2}$ for C=0 (middle) and *C* = 2 (right). The graph has been generated using an alphabet with 10 symbols so all the points of the graph are inside the decagon drawn in red.

**Figure 8:**
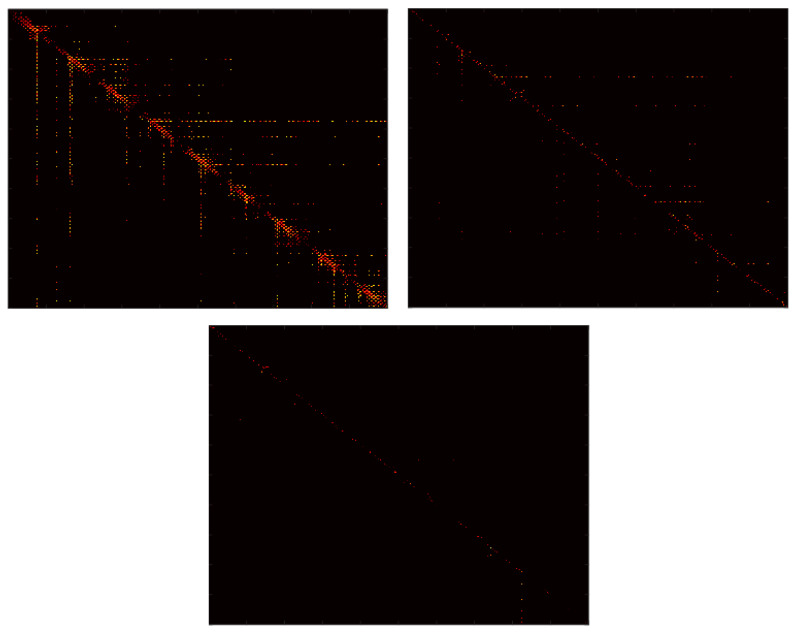
WAM images for C=0 (top-left), C=0.5 (top-right) and C=1 (bottom).

The CGRS images and graphs have been constructed using a SAX algorithm with a 10 characters alphabet. An illustration is presented in Figure 7. Both SSIM and SNESV evolutions become sensitive to the increase of the coupling factor after the threshold C>0.8, but with significant oscillations. The graph representation seems to be more sensitive as the range of variation of SNSEV values becomes larger. In the case of complex networks, the evolution of the entropy $E_{WAM}$ is almost monotonic for the whole range of variation of C, with no saturation effects. The evolution of the WAM images is illustrated in Figure 8. When representing time series by cross-visibility networks, for low coupling, the reciprocal visibility between two bars is frequently limited by the obstacles created by the other time series. For stronger coupling, the time series becomes increasingly synchronized and the WAM image evolves into a less random structure, characterized by lower entropy.

### 3.2. Experiments

The discharges considered for this study are the same as in [7] in order to allow a comparison of the methods analyzed in these two papers. For these pulses, covering both L and H confinement modes, a central hydrogen minority ICRH, in the range of 3–4 MW with a modulation frequency of f = 5 Hz, was used and the H-mode discharges had approximately 10 MW of NBI applied to the plasma. 

**Figure 9:**
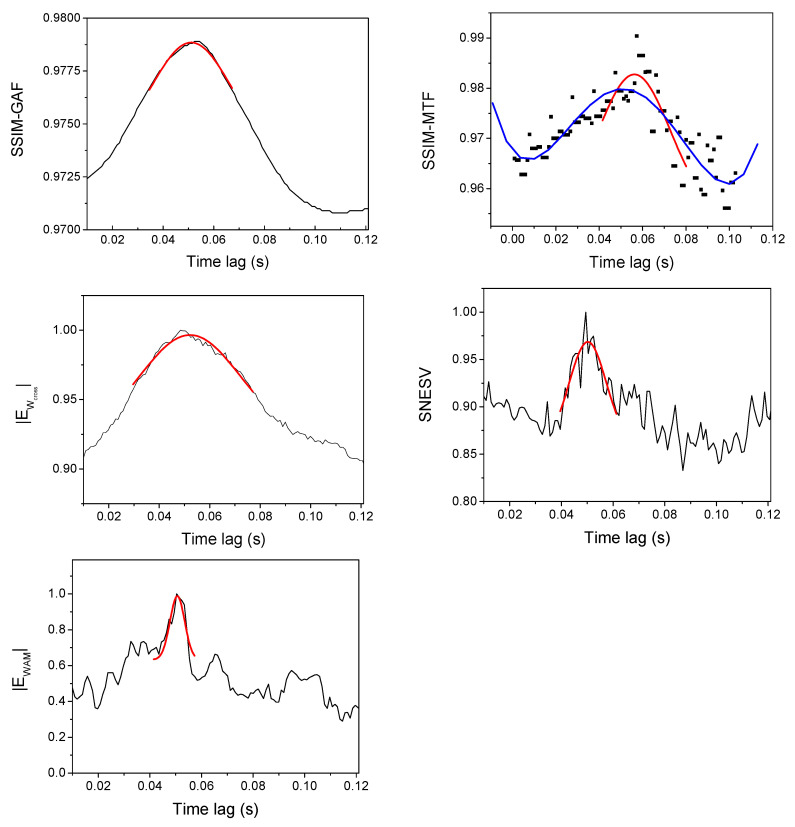
The time-lagged evolution of the coupling measures for the JET discharge #89826: SSIM calculated for GAF images (top-left), SSIM calculated for MTF images (top-right), absolute value of the CMM $W_{cross}$ image entropy (middle-left), SNSEV entropy for the CGRS graphs (middle-right) and the absolute values of the WAM image entropy (bottom-left). The absolute values of the entropies (SNSEV, $E_{cross}$ and $E_{WAM}$) are normalized to their maximum value. The Gaussian fit of the peaks is reported in red. For the case of SSIM-MTF an alternative fit (reported in blue) has been performed using a 4-th order polynomial and all the points of the plot.

**Figure 10:**
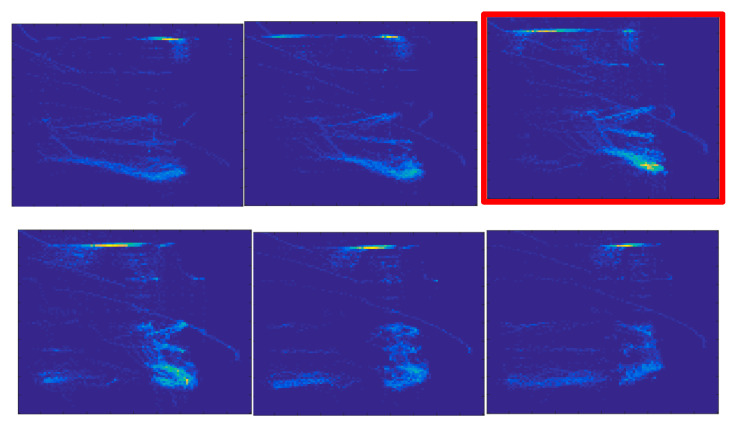
The evolution of the $W_{cross}$ images for different time lag values. The top row shows the images for t=0 ms (left) and t=0.025 ms (middle) and t=0.052  ms. The bottom row shows the values for t=0.075  ms (left), t=0.100  ms (middle) and *t* = 0.120 ms (right). The image for which the absolute value of *W_cross_* entropy reaches its maximum is marked by a red border.

For each pulse, the time lag between the ICRH power and central electron temperature time series has been varied in the interval [0, 120] ms, in steps of 1 ms. The variation of each coupling measure has been recorded and the position of the main peak has been used to determine the time lag for which the pacing reaches its maximum efficiency. An illustrative example is presented in Figure 9 for JET pulse #89826. GAF and CMM methods capture the main tendency in the evolution of the coupling between the two time series providing a smooth curve. SNSEV and WAM profiles show various oscillations. The position of the main peak corresponds with those given by GAF and CMM methods. The MTF curve is characterized by significant spurious oscillations and the determination of the position of the main peak is ambiguous. In Figure 9, two different positions have been obtained when fitting the points located around the maximum values with a Gaussian or fitting the whole curve with a four-degree polynomial. In the case of the CMM method, introduced in this paper, Figure 10 shows the evolution of the $W_{cross}$ image for various values of the time lag. The image corresponding to the time lag for which the $W_{cross}$ entropy has its maximum absolute value marked by a red border and it clearly shows a more structured and sharper form. 

The observations made for the JET pulse #89826 remain valid in general also for the other three pulses included in this study: #89822, #90005 and #90006. The time lag values, for which the various indicators reach their maximum, are listed in Table 1. This table shows also the values obtained with the methods reported in [7]. GAF, CMM and CN estimates are in good agreement with these values. Only in one case, for each method, the time lag value corresponding to the maximal coupling lies outside the interval given by the confidence interval reported in [7] but the difference is only of 2 ms (approx. 4%). In the case of the CGRS method, the differences increase by up to 8%. For the MTF method, the discrepancy increases significantly. 

The present estimates can be compared also to the slowing-down time of the ions, shown in the last column in Table 1; the large uncertainty of these values reflect the strong variations of the central electron temperature during a sawtooth cycle. It can be observed that the causal influence of the ICRH on the sawteeth is estimated to occur in a time period, which agrees very well with the slowing-down time of the ions. This is in favor of the interpretation that the main factor in the pacing scheme is the stabilization effect of the fast particles.

## 4. Conclusions

A typical difficulty of the experiments, aimed at sawtooth pacing by modulating the ICRH power, is their reliable interpretation. The evaluation of the number of sawteeth effectively triggered by the intentional ICRH power modulations is not a simple task, since sawteeth are quasiperiodic and, therefore, if enough time is allowed to elapse, they would occur almost always after a notch in the RF. The determination, on the basis of time series analysis, of the time horizon over which the ICRH power modulations are effective in triggering the instabilities, could be a solution to this problem. 

In this paper, a group of methods, based on the image representation of time series, has been introduced for the assessment of the pacing technique. A series of numerical tests have shown the potential of the three techniques to determine the time interval over which a causal-effect relationship takes place. The application to JET experiments shows that three methods give quite coherent results and in good agreement with the values obtained by other techniques, based on different principles [7]: the method based on the Gramian Angular Field representation; a variation of the Markov transition matrix defined for a pair of time series; a method which uses the time series transformation into a cross-visibility network whose adjacency matrix is represented as an image. The approach based on the chaos game representation has the advantage of a fast computation but its confidence intervals are larger. The small discrepancies between the estimates of the various methods can be interpreted as the confidence intervals in the results. From a physical point of view, the methods support the interpretation that the fast ions play a fundamental role in the stabilization of the sawteeth, in both L and H mode.

Significant future work will be dedicated to testing the applicability of these methods to other coupled dynamical systems, involving different complicated phenomena and characterized by full chaotic behavior. Sophisticated techniques for the generation of numerical time series [45], and applications coming from various domains (e.g., climatology, financial, etc.), should be addressed in order to obtain a comprehensive understanding of the efficiency of these methods. 

## Figures and Tables

**Table 1:** Time lag values for maximum coupling between the ICRH and electron temperature time series. The first column reports the shot number and the second the confinement regime (L or H). For each indicator, the following columns provide the estimated time corresponding to the maximum causal influence. The penultimate column shows the values’ interval reported in [7]. The last column reports the slowing-down time of the minority (hydrogen) ions. When the values of the indicators lie in the interval reported in [7] they are marked by bold numbers. The uncertain values and the values located outside the confidence interval of the ions’ slowing-down times are marked with italic numbers.

Pulse Number	Regime	GAF (ms)	MTF (ms)	CMM (ms)	CGRS (ms)	CN (ms)	Time of Maximum Causal Influence Reported in [7] (ms)	Slowing-Down Time of the Ions (ms)
**89822**	L	**52**	57	**52**	48	50	[52, 54]	50 (+/−10)
**89826**	L	**51**	50/56	**52**	**50**	**51**	[52, 54]	50 (+/−10)
**90005**	H	74	96	73	64	**70**	[68, 72]	80 (+/−20)
**90006**	H	**85**	102/108	**87**	81	**89**	[85, 95]	80 (+/−20)
